# Associations between selective serotonin reuptake inhibitors and adverse events following hip fracture arthroplasty: a retrospective cohort study

**DOI:** 10.1177/11207000251374546

**Published:** 2025-12-18

**Authors:** Lynn Lethbridge, Matt Nagle, Emily Johnson, C Glen Richardson, Michael J Dunbar

**Affiliations:** 1 Department of Surgery, Dalhousie University, Halifax, Nova Scotia, Canada; 2 University of Limerick Hospital Group, Dublin, Ireland; 3 Nova Scotia Health Authority, Halifax, Nova Scotia, Canada

**Keywords:** Arthroplasty, fracture, patient outcomes, SSRI

## Abstract

**Background:**

Hip fractures are a priority topic while selective serotonin reuptake inhibitors (SSRI) use is increasing. Surgical outcomes over longer follow-up periods for hip fracture patients on SSRIs is unclear. The purpose of this study was to test for associations between SSRIs and post-surgical adverse events for hip fracture arthroplasty patients.

**Methods:**

Hospital data were used to select patients who had hip fracture arthroplasty surgery in Nova Scotia, Canada from 2016 to 2022. Patients who filled an SSRI prescription (Rx) in the 180-day period prior to surgery were identified. Study outcomes were any emergency department (ED) visit, mortality, revision, and major bleeding within 180 days of discharge as well as a blood transfusion during admission. Multivariate hierarchical logistic models weighted by inverse probability treatment weights were estimated to test for associations between SSRI use and outcomes.

**Results:**

An SSRI prescription was filled in the 180-day pre-surgery period for (883) 29.9% of the 2946 cases. Adjusted odds ratios were higher for those on an SSRI for an ED visit (1.68 CI, 1.40–2.01; *p* < 0.0001), mortality (1.26 CI, 1.02–1.55; *p* = 0.036), revision (2.35 CI, 1.36–4.06; *p* = 0.0022), and bleeding event (1.48 CI 1.06–2.07; *p* = 0.022). Blood transfusion was statistically insignificant.

**Discussion:**

SSRI use was associated with worse outcomes for hip fracture patients for four of five study outcomes. SSRI use should be discussed prior to surgery to mitigate the likelihood of adverse events.

## Introduction

Hip fractures are a priority topic globally due to the high mortality rates and long-term impairment of activities.^1,2^ Along with the devastating effect on patients, there is a considerable burden on the health care system following a fracture.^3,4^ The risk of a hip fracture increases with age for both men and women,^5,6^ consequently, with ageing populations, it is expected that the number of hip fractures will increase putting more stress on patients, families and health care systems.

Those with a hip fracture are a population susceptible to depression as the literature has shown an association between depression and hip fractures.^7,8^ Antidepressant medications include selective serotonin reuptake inhibitors (SSRIs) which are the most commonly prescribed type of antidepressant and are increasingly used for anxiety, pain, insomnia, minor depressive symptoms.^9,10^ SSRIs have been shown to be associated with falls^11^ as side effects include dizziness, fatigue, cognitive function and vision problems.^12,13^ SSRIs are also associated with lower bone mineral density.^14,15^ These factors combine to worsen the consequences for an already vulnerable population.

Individuals on SSRIs are more likely to experience negative outcomes following many types of surgery which includes excess bleeding, infection, and mortality.^16–18^ Research has shown hip fracture arthroplasty patients on SSRIs have an increased likelihood of adverse events, with the follow-up period often being 30 or 90 days after surgery.^19–23^ Recovery from a hip fracture, however, has been shown to take 6–12 months or longer.^2,24–27^ There is a paucity of studies analysing a broad range of hip fracture surgical outcomes for those on SSRIs for a longer follow-up period despite the prolonged recovery period. The purpose of this study was to test for associations between SSRIs and post-surgical adverse events for hip fracture arthroplasty patients during the 180-day period following surgery. Unadjusted and adjusted outcomes for those on SSRIs prior to surgery were compared to those who were not on SSRIs.

## Methods

The population for this retrospective cohort study was selected from hospital discharge data, specifically the Discharge Abstract Database (DAD) administered by the Canadian Institutes for Health Information (CIHI). Physician claims, patient registry, and Drug Information System (DIS) data were linked to the study cohort which provided a comprehensive health profile for all cases before and after surgery. The National Prescription Drug Utilization Information System (NPDUI), a listing of drug information database administered by the CIHI, was used to select SSRIs.

### Cohort

All observations with an International Classification of Disease -version 10 (ICD-10) code starting ‘S72’, defined as ‘fracture of the femur’(WHO icd-10 source), upon admission from October 2016 to September 2022 in Nova Scotia (NS), Canada were selected for inclusion in the study population. The diagnostic coding is a broad definition of fracture since intracapsular neck fracture, the type normally treated with arthroplasty’ does not have a specific ICD-10 coding. Canadian Classification of Interventions procedure codes ‘1VA53’ and ‘1SQ53’, joint replacement of the hip and pelvis respectively, were used to identify arthroplasty cases. Observations without a NS residential postal code were excluded since pre- and post-surgical health utilisation data were not available for these cases.

### Exposure

The study exposure was any SSRI prescription (Rx) filled in the 180-day period prior to surgery as identified from the DIS, a database which includes all prescriptions filled at pharmacies in NS. SSRIs were identified from the NPDUI data, namely citalopram, escitalopram, fluoxetine, fluvoxamine, paroxetine, sertraline, vilazodone, and vortioxetine, and linked to the DIS database through a common drug identifier number.

### Outcomes

The study outcomes were any Emergency Department (ED) visit, mortality, implant revision surgery, and a major bleeding event occurring in the 180-day period after surgical discharge, each analysed separately. ICD-9 and ICD-10 codes were used to identify a bleeding event from the physician claims and hospital data. A broad range of bleeding codes were utilized following Yokoyama et al.^28^ A blood transfusion administered during surgical admission was also modelled.

### Covariates

Covariates were selected based on clinical expertise, previous literature and data availability.^16,19^ Patient characteristics included were age, sex, distance to surgical hospital from residence, a binary indicator of admission from home and co-morbidities. The Charlson Comorbidity Index (CCI)^29^ was used as the basis for included health conditions. Each health condition from the CCI was included in the probability of treatment model except AIDS/HIV which was excluded for confidentiality reasons. Additional study-specific conditions included were osteoarthritis, anemia and depression. Diagnostic codes from hospital and physician claims billings were used to identify each comorbidity based on the Canadian Chronic Disease Surveillance System (CCDSS) which uses multiple data sources and algorithms developed by multidisciplinary expert input.^30^ For the outcome models, an aggregate variable of co-morbidities was generated and binary variables indicating one co-morbidity and 2 or more co-morbidities was included in the regressions with zero being the reference category. Individual disease indicators were not used for outcome regressions due to low event numbers however the aggregate Charlson score was included instead. To control for temporal changes and hospital conditions, year of surgery and hospital were also included as covariates.

### Statistical analysis

2-level hierarchical models were estimated to test for associations between SSRI use and study outcomes where individuals were the first level and the five hospitals which carry out hip arthroplasty in the province were the second level. To minimise treatment selection bias of those who take SSRIs, Inverse Probability of Treatment Weights (IPTW) were generated. IPTW is a propensity score method which best estimates average treatment effect across the entire population and not just those treated.^31^ To minimise disproportionate influence of extreme weights, stabilised weights were generated whereby the numerator for the exposed group is replaced by the proportion treated and for the unexposed, the proportion not treated.^32^ Crude standardised differences (SDs) between groups were generated and compared to IPTW SDs to check for balancing of characteristics after weighting. To test for associations between SSRI use and study outcomes, we ran logistic regressions weighted by the stabilised IPTW with controls for confounders which are known to affect outcomes. Inverse probability weighted regression adjustment models (IPWRA) odds ratios (ORs) from each regression were generated for all outcomes. A 95% confidence level (CI) was chosen as the significance threshold with no adjustment for multiple tests since an omnibus null hypothesis was not being tested.^33^

Ethics was granted by the Nova Scotia Health Authority and all analysis was carried out using SAS 9.4.

## Results

There were 2946 hip fracture arthroplasty cases, of which 2099 (71.2%) were female and the overall average age was 80.1 (SD 10.3) years. The study population included 2715 (92.1%) individuals which had at least one co-morbidity and the mean neighbourhood median household income was $Can 70,396 (SD 21,300). There were 883 (30.0%) who had a SSRI Rx filled in the 180-day period prior to surgery. For those with a prior SSRI, 474 (53.7%) Rxs were refills an indication that just over half had longer term use.

To check for balance between the treated and untreated groups after IPTW weighting, Table 1 shows the unweighted and weighted counts for the study covariates as well as the absolute standardised differences. ORs for the hierarchical logistic regression models used to generate the propensity scores for the IPTW weights are shown in Supplemental Table 1. The largest differences for the unweighted results occur for depression and dementia. After the IPTW adjustment, the absolute standardised differences for all co-variates are less than 10, the threshold commonly used the indicate effective balance.^32^

**Table 1.** Covariate Comparison before and After IPTW Adjustment.

Variable	Before IPTW Adjustment Count (%)			After IPTW Adjustment Count (%)		
	No SSRI 180 prior	SSRI 180 prior	Absolute Standardised Difference	No SSRI 180 prior	SSRI 180 prior	Absolute Standardised Difference
**Year of surgery 2016**	63 (3.05)	31 (3.51)	1.80	70.5 (3.37)	27.0 (3.14)	0.92
**Year of surgery 2017**	308 (14.93)	111 (12.57)	4.66	298.6 (14.26)	130.5 (15.17)	1.73
**Year of surgery 2018**	341 (16.53)	146 (16.53)	0.01	343.4 (16.40)	143.4 (16.67)	0.49
**Year of surgery 2019**	372 (18.03)	133 (15.06)	5.39	361.7 (17.28)	140.3 (16.310	1.74
**Year of surgery 2020**	371 (17.98)	147 (16.65)	2.38	363.3 (17.35)	147.7 (17.17)	0.33
**Year of surgery 2021**	307 (14.88)	176 (19.93)	8.97	346.8 (16.57)	144.9 (16.85)	0.51
**Year of surgery 2022**	301 (14.59)	139 (15.74)	2.17	309.4 (14.78)	126.5 (14.70)	0.14
**Hospital 1**	419 (20.31)	197 (22.31)	3.24	429.2 (20.50)	189.8 (22.07)	2.54
**Hospital 2**	229 (11.10)	94 (10.65)	1.00	221.9 (10.60)	80.8 (9.39)	2.76
**Hospital 3**	352 (17.06)	155 (17.55)	0.87	367.6 (17.56)	163.0 (18.95)	2.43
**Hospital 4**	616 (29.86)	243 (27.52)	3.34	614.4 (29.34)	232.6 (27.05)	3.30
**Hospital 5**	447 (21.67)	194 (21.97)	0.49	460.7 (22.00)	193.9 (22.54)	0.85
**Age** < **65 years**	164 (7.95)	73 (8.27)	0.81	183.3 (8.75)	76.2 (8.86)	0.25
**Age 65–84 years**	1124 (54.48)	472 (53.45)	1.16	1126.7 (53.81)	448.5 (52.13)	1.90
**Age** ⩾ **85 years**	775 (37.57)	338 (38.28)	0.91	783.8 (37.43)	335.6 (39.01)	2.00
**Female**	1429 (69.27)	670 (75.88)	6.88	1495.4 (71.42)	630.7 (73.32)	1.97
**Diabetes with complications**	481 (23.32)	209 (23.67)	0.55	507.5 (24.24)	218.6 (25.41)	1.77
**Diabetes without complications**	274 (13.28)	126 (14.27)	1.95	311.2 (14.86)	132.8 (15.44)	1.09
**Myocardial infarction**	57 (2.76)	22 (2.49)	1.19	54.8 (2.62)	26.6 (3.09)	2.02
**Heart failure**	217 (10.52)	95 (10.76)	0.53	238.7 (11.40)	98.9 (11.50)	0.22
**Ischemic heart disease**	329 (15.95)	144 (16.31)	0.66	333.6 (15.93)	145.1 (16.87)	1.70
**Osteoporosis**	579 (28.07)	268 (30.35)	3.24	601.9 (28.75)	248.7 (28.91)	0.24
**Periphral vascular disease**	111 (5.38)	33 (3.74)	5.51	104.3 (4.98)	45.0 (5.23)	0.78
**Cerebral vascular disease**	162 (7.85)	89 (10.08)	5.38	198.4 (9.470	70.4 (8.18)	3.15
**Chronic pulmonary disease**	328 (15.90)	161 (18.23)	4.18	352.8 (16.85)	152.8 (17.76)	1.62
**Rheumatic disease**	98 (4.75)	33 (3.74)	3.52	91.8 (4.39)	39.6 (4.60)	0.72
**Peptic ulcer**	39 (1.89)	12 (1.36)	2.96	35.9 (1.71)	16.6 (1.93)	1.14
**Mild liver disease**	29 (1.41)	18 (2.04)	3.42	31.9 (1.53)	12.5 (1.45)	0.43
**Paraplegia and hemiplegia**	36 (1.75)	30 (3.40)	7.34	61.7 (2.95)	20.4 (2.37)	2.52
**Renal disease**	158 (7.66)	69 (7.81)	0.40	157.3 (7.51)	63.6 (7.40)	0.31
**Cancer**	287 (13.91)	100 (11.33)	5.32	282.4 (13.49)	109.0 (12.68)	1.64
**Moderate/sever liver disease**	11 (0.53)	7 (0.79)	2.26	12.9 (0.61)	5.1 (0.59)	0.18
**Metastatic carcinoma**	64 (3.10)	27 (3.06)	0.18	69.6 (3.32)	26.6 (3.09)	0.92
**Osteoarthritis**	883 (42.80)	404 (45.75)	3.55	913.1 (43.61)	367.8 (42.75)	1.04
**Anaemia**	540 (26.18)	294 (33.30)	10.02	598.9 (28.60)	244.7 (28.44)	0.23
**Dementia**	371 (17.98)	348 (39.41)	30.92	540.8 (25.83)	221.8 (25.78)	0.07
**Depression**	258 (12.51)	430 (48.70)	51.93	515.2 (24.61)	208.8 (24.27)	0.51
**Median neigbourhood income** < **25th percentile**	523 (25.35)	204 (23.10)	3.45	516.3 (24.66)	221.5 (25.75)	1.64
**Median neigbourhood income** > **75th percentile**	508 (24.62)	228 (25.82)	1.80	516.7 (24.68)	211.8 (24.62)	0.08
**Lives** < **20 kms from surgical hospital**	1085 (52.59)	465 (52.66)	0.08	1083.3 (51.74)	449.5 (52.26)	0.59
**Lives 20-49 kms from surgical hospital**	205 (9.94)	91 (10.31)	0.84	217.8 (10.40)	88.5 (10.29)	0.26
**Lives 50-100 kms from surgical hospital**	483 (23.41)	196 (22.20)	1.91	480.6 (22.96)	199.8 (23.23)	0.43
**Lives** > **100 kms from surgical hospital**	290 (14.06)	131 (14.84)	1.50	312.0 (14.90)	122.3 (14.22)	1.31
**Lives in a rural area**	672 (32.57)	282 (31.94)	0.87	676.2 (32.30)	275.0 (31.98)	0.44

### Outcomes

All study outcome point estimate percentages were higher for those who filled an SSRIs Rx in the 180-day period prior to surgery both before and after adjustments for confounders (Table 2). Focusing on the adjusted results, 31.2% (275.5/883) versus 21.3% (440.4/2063) had an ED visit, 20.5% (180.9/883) versus 17.0% (351.7/2063) died, 0.86% (7.6/883) versus 0.37% (7.7/2063) had a revision within the 180-day follow-up period, 5.9% (51.9/883) versus 5.4% (111.6/2063) had a transfusion during admission, and 4.5% (39.8/883) versus 3.1% (63.8/2063) a bleeding event.

**Table 2.** Unadjusted and IPTW RA counts and rates for study outcomes.

	Unadjusted		IPTWR Adjusted^a^	
	No SSRI 180 prior	SSRI 180 prior	No SSRI 180 prior	SSRI 180 prior
	Count (%)	Count (%)	Count (%)	Count (%)
**Emergency Department visit**	481 (23.3)	263 (29.8)	440.4 (21.3)	275.5 (31.2)
**Mortality**	324 (15.7)	181 (20.5)	351.7 (17.0)	180.9 (20.5)
**Revision**	30 (1.5)	20 (2.3)	7.7 (0.37)	7.6 (0.86)
**Blood transfusion during surgical admission**	183 (8.9)	93 (10.5)	111.6 (5.4)	51.9 (5.9)
**Bleeding event**	101 (4.9)	47 (5.3)	63.8 (3.1)	39.8 (4.5)
**Observations**	2063	883	2063	883

a Odds ratios for covariates given in Supplemental Tables 1–10.

**Figure 1.**
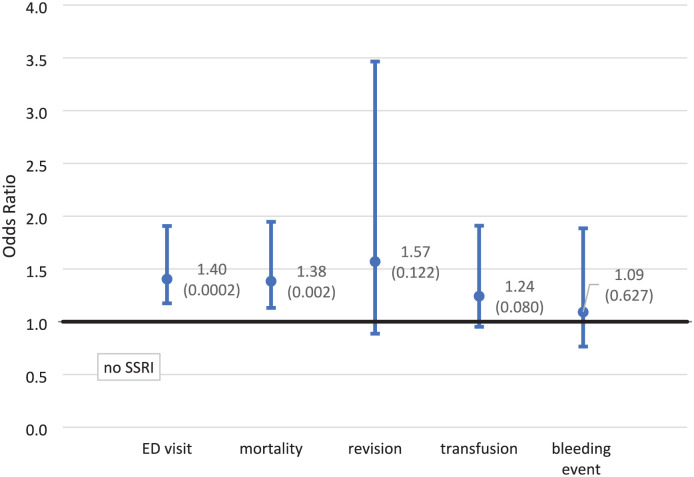
Association between SSRI use prior to surgery and study outcomes unadjusted odds ratios (*p*-values). Note: confidence intervals with 95% confidence.

**Figure 2.**
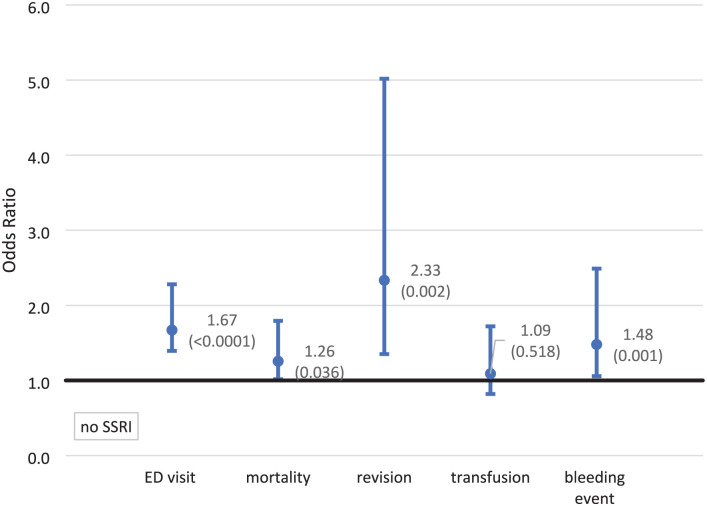
Association between SSRI use prior to surgery and study outcomes IPTWR adjusted odds ratios (*p*-values). Note: confidence intervals with 95% confidence.

The unadjusted outcome ORs comparing those who had an SSRI Rx and those who did not are 1.40 (1.18–1.68) and 1.38 (1.13–1.69) for an ED visit and mortality. The remaining outcomes were not statistically different (Figure 1). After the IPTWRA, all ORs were higher and significant except for blood transfusion which was statistically insignificant. The adjusted ORs were 1.67 (1.39–2.00) for an ED visit, 1.26 (1.02–1.55) for mortality, 2.33 (1.35–4.04) for a revision, and 1.48 (1.06–2.07) for a bleeding event. A blood transfusion during admission remained statistically insignificant (Figure 2). Of the study outcomes, the highest IPTWRA OR was for a revision which was 2.33 (1.35–4.04) times more likely for those on an SSRI prior to surgery.

### Sensitivity

To test the sensitivity of our results, we ran the models using 90-day outcomes, a common follow-up length found in the literature. The IPTWRA results yielded an OR of 1.65 (1.35–2.03) for an ED visit, and revision 2.68 (1.47–4.90). Mortality and bleeding were not statistically significant.

## Discussion

Our study indicated an association between SSRI use prior to arthroplasty surgery for hip fracture patients and negative outcomes for 4 of 5 outcomes for the 6-month period following surgery. SSRIs have been shown to be associated with an increased risk of hip fracture although the exact mechanism remains unclear.^34,35^ SSRIs may increase the likelihood of falls and may affect bone health,^36,37^ so it is plausible SSRI use contributed to the initial hip fracture in our study population. Our results suggest that SSRIs may have further detrimental effects among hip fracture patients treated with arthroplasty surgery. Bone healing may be affected by SSRI use.^38^ As well, there is some evidence, irrespective of SSRI use, that bone mineral density and muscle loss continues to decrease one year after the fracture,^26^ suggesting this population is particularly susceptible to long-term adverse events. Our results are consistent with this literature.

Hip fracture patients often suffer from a broad range of medical, mobility, and functional impairments.^24,39,40^ Outcomes in our study, ED visits, mortality, revision and bleeding events are critical outcomes studied in hip fracture literature^41,42^ and include a comprehensive range of adverse events when analysed together. The outcome with largest effect size is revision surgery with the odds of those on an SSRI being over twice that of those who were not. The unadjusted revision rate was 2.3% while the adjusted rate was 0.84% for those on SSRIs which compares to a rate of 0.04%-0.05% for revisions generally in a recent systematic review^41^. Bruun et al^19^ reported a re-operation 30-day rate of 5.5% for SSRI users compared to 4.5% for non-users among hip fracture arthroplasty patients which is a lower rate ratio than our study (1.22 vs 1.53). In the Bruun et al. study,^19^ however, the follow-up period is shorter and re-operation includes a larger range of surgeries than revision alone. That same study reported a 30-day mortality of 13.0% for SSRI users and 9.9% for non-users for a ratio of 1.31 which compares to our 180-day rates of 20.5% and 15.7% for a comparable rate ratio of 1.30.

A transfusion during admission was the only outcome to show no statistically significant association with SSRI use. Hoveidaei et al.^43^ found SSRI users had a significantly higher rate of blood transfusion in hip fracture patients although SSRI use was only tracked in the 30-day period prior to surgery. A recent study by Fortier et al^44^ reported a difference in the likelihood of a transfusion in the 1–7 day follow-up period between current SSRI users and non-SSRI users but no difference in the same day or 7–30 day period rates. In our study, unlike transfusions, a bleeding event in the six-month period following discharge was statistically significant and more likely for those on SSRIs. Van Haelst et al.^45^ found a higher mean level of blood loss during surgery for orthopaedic patients on SSRIs but the level was not enough to require a transfusion. It is possible that SSRI patients in our study experienced higher levels of blood loss during the surgery, but not enough to require a transfusion. Blood loss amounts were not available for our study.

The follow-up period studied in hip fracture arthroplasty research is often shorter than the time it takes to fully recover from the injury. As surgery normally occurs within a few days of the fracture, this study of arthroplasty patients helps fill a gap in the literature by analysing effects over a period more in line with recovery time. Sensitivity analysis showed a 90-day follow-up had statistically significant association between SSRI use and ED visits as well as revision surgery but mortality and bleeding events were insignificant. Inconsistent results for mortality are of particular concern as mortality rates for hip fracture patients are relatively high in general and is an outcome which is frequently studied. Of those who died within 6 months of surgery, a lower percentage occurred in the first 90 days for those with previous SSRI use (135/181 [74.5%]) compared to those without SSRI (262/324 [80.9%]) use suggesting a longer follow-up time is even more important for those with previous SSRI use as information may be missed. Finally, unadjusted ORs were statistically insignificant for revision and a bleeding event but significantly higher for those on SSRIs once adjustments were incorporated highlighting the importance of controlling for confounders.

Our study results indicate a link between SSRI use and negative outcomes after hip fracture arthroplasty, however, research has shown depression itself to be a risk factor for a bone fracture.^46,47^ Disentangling the effects of depression and antidepressant use is complicated as side effects are similar. Factors such as mood disorders, functional limitations,^16^ cognitive dysfunction and falls are more likely in those with depression which can contribute to poor surgical outcomes.^9,48^ A lack of information on a diagnosis of mental illness from any source or only from hospital data has been noted as a limitation in previous SSRI research.^19,49^ Controlling for a depression diagnosis which includes both hospital and primary care source in both the IPTW and as a covariate is a strength of this study. Not all hip fracture patients are treated surgically. Our study does not examine associations between SSRIs on the probability of a hip fracture, rather compares outcomes for those who did have surgery. Risk factors due to SSRIs for hip fractures are likely similar to negative outcomes among those who had surgery. Finally, the study population includes both those who were on SSRIs for a relatively shorter time periods and those who had were treated for a longer period as measured by the percentage with a refill Rx. Outcome results are similar between the groups (data not shown).

### Limitations

Our study has several limitations. The weaknesses of administrative data have been highlighted in the literature.^50^ Most notably the use of diagnostic codes may result in inaccuracies particularly for health conditions that require a more rigorous set of rules to identify. More specific measures for frailty which could be included as a confounder would strengthen the results. The use of case definitions based on the CCDSS for co-morbidities will help to mitigate this limitation.^30^ Additionally, there is a lack of detail in the administrative data sources to determine the reason for the revision which could more precisely target the effect of SSRIs. To identify hip fracture arthroplasty cases, both diagnostic and procedure codes were used which reduces the chance of inaccuracies. As noted, recovery from hip fracture arthroplasty can take a year or longer, so adverse events could occur after the 180-day follow-up period used in this study. ED visits include all visits whether medical or surgical for medical or surgical reasons. There is a lack of information in our administrative data to accurately categorise into medical or surgical reasons. The effect of perioperative SSRIs is not accounted for in our study as we did not have access to drugs administered from hospital pharmacies. Auerbach et al.^16^ showed SSRIs administered perioperatively adversely affected outcomes for patients who underwent major surgery. It is unclear if those who had an SSRI Rx prior to surgery were more or less likely to receive one during admission or how that may have affected overall results. Finally, we do not have information on medication adherence after the Rx is filled. A patient may have had a Rx filled but did not take the medication as prescribed which may have affected our findings.

## Conclusion

Our study suggests prior use of SSRIs before hip arthroplasty surgery is associated with a higher probability of adverse events in the 180-day follow-up period. Surgeons should discuss SSRI use with patients prior to surgery to mitigate the likelihood of adverse events during follow-up.

Future studies should include prospective data collection to obtain targeted information on the hip fracture population.

## Supplemental Material

sj-pdf-1-hpi-10.1177_11207000251374546 – Supplemental material for Associations between selective serotonin reuptake inhibitors and adverse events following hip fracture arthroplasty: a retrospective cohort study Supplemental material, sj-pdf-1-hpi-10.1177_11207000251374546 for Associations between selective serotonin reuptake inhibitors and adverse events following hip fracture arthroplasty: a retrospective cohort study by Lynn Lethbridge, Matt Nagle, Emily Johnson, C Glen Richardson and Michael J Dunbar in HIP International
