# Supplementary material for: Associations between selective serotonin reuptake inhibitors and adverse events following hip fracture arthroplasty: a retrospective cohort study
Source: Hip Int. 2025 Dec 18;36(1):125–34. doi: 10.1177/11207000251374546 (PMC12876436; doi:10.1177/11207000251374546)
Supplement: sj-pdf-1-hpi-10.1177_11207000251374546 – Supplemental material for Associations between selective serotonin reuptake inhibitors and adverse events following hip fracture arthroplasty: a retrospective cohort study [file sj-pdf-1-hpi-10.1177_11207000251374546.pdf]

Supplemental Table 1: Odds Ratios Probability of an SSRI in 180 Period prior to Surgery

	Odds Ratio	Lower CI	Upper CI	p-value
**Reference year 2016**				
Year of surgery 2017	0.781	0.453	1.349	0.3758
Year of surgery 2018	0.987	0.577	1.687	0.9609
Year of surgery 2019	0.801	0.467	1.373	0.4193
Year of surgery 2020	0.850	0.498	1.453	0.5532
Year of surgery 2021	1.311	0.769	2.238	0.3199
Year of surgery 2022	1.097	0.639	1.885	0.737
**Reference age 65-84**				
Age < 65	1.209	0.858	1.704	0.2784
Age >= 85	1.060	0.873	1.289	0.5551
**Reference male**				
Female	1.345	1.089	1.662	0.006
**Reference not diagnosed with condition**				
Diabetes with complications	1.014	0.781	1.317	0.9155
Diabetes without complications	1.104	0.785	1.551	0.5700
Myocardial infarction	0.82	0.447	1.507	0.5234
Heart failure	1.001	0.731	1.37	0.9957
Ischemic heart disease	1.132	0.859	1.493	0.3772
Osteoporosis	1.021	0.833	1.251	0.844
Peripheral Vascular Disease	0.625	0.394	0.992	0.0462
Cerebral vascular disease	1.144	0.811	1.613	0.4446
Chronic Pulmonary Disease	1.327	1.039	1.693	0.0232
Rheumatic disease	0.642	0.407	1.013	0.0571
Peptic ulcer	0.509	0.242	1.071	0.0752
Mild liver disease	1.315	0.609	2.843	0.4857
Paraplegia and Hemiplegia	2.127	1.154	3.918	0.0155
Renal disease	1.064	0.756	1.497	0.7214
Cancer	0.806	0.592	1.096	0.1686
Moderate/sever liver disease	1.652	0.512	5.326	0.4008
Metastatic Carcinoma	1.382	0.769	2.486	0.2795
Osteoarthritis	1.061	0.884	1.274	0.5241
Anemia	1.312	1.073	1.605	0.0083
Dementia	3.039	2.484	3.719	<.0001
Depression	6.603	5.416	8.05	<.0001
**Reference group median neigbourhood income 25th-75th percentile**				
Median neigbourhood income < 25th percentile	0.917	0.733	1.147	0.4474
Median neigbourhood income > 75th percentile	1.070	0.851	1.344	0.5632
**Reference distance from surgical hospital <=19**				

Lives 20-49 kms from surgical hospital	1.246	0.898	1.729	0.1876
Lives 50-100 kms from surgical hospital	0.926	0.695	1.235	0.6010
Lives > 100 kms from surgical hospital	1.164	0.846	1.601	0.3506
**Reference lives in urban area**				
Lives in a rural area	1.042	0.822	1.319	0.736

Supplemental Table 2: Odds Ratios Probability of an Emergency Department Visit within 180 Days

	Odds Ratio	Lower CI	Upper CI	p-value
**Reference group no SSRI Rx in 180-day period prior to surgery**				
SSRI RX 180 days prior to surgery	1.671	1.394	2.003	<.0001
**Reference year 2016**				
Year of surgery 2017	0.944	0.699	1.275	0.7059
Year of surgery 2018	1.207	0.906	1.608	0.1992
Year of surgery 2019	0.93	0.696	1.244	0.6268
Year of surgery 2020	1.126	0.848	1.495	0.4117
Year of surgery 2021	0.872	0.649	1.17	0.3607
**Reference age 65-84**				
Age < 65	1.064	0.783	1.444	0.693
Age >= 85	0.877	0.727	1.056	0.1662
**Reference male**				
Female	0.891	0.738	1.077	0.2346
**Reference not diagnosed with condition**				
One Charlson co-morbidity	1.491	1.066	2.084	0.0195
Two or more Charlson co-morbidities	2.275	1.667	3.106	<.0001
Dementia diagnosis	0.546	0.44	0.679	<.0001
Depression diagnosis	1.069	0.872	1.311	0.5197
Anemia diagnosis	0.928	0.761	1.132	0.4622
**Reference group median neigbourhood income 25th-75th percentile**				
Median neigbourhood income < 25th percentile	1.073	0.870	1.325	0.5087
Median neigbourhood income > 75th percentile	0.904	0.727	1.123	0.3614
**Reference distance from surgical hospital <=19**				
Lives 20-49 kms from surgical hospital	0.916	0.665	1.262	0.592
Lives 50-100 kms from surgical hospital	0.720	0.546	0.949	0.0195
Lives > 100 kms from surgical hospital	0.919	0.677	1.248	0.5872
**Reference lives in urban area**				
Lives in a rural area	0.554	0.439	0.698	<.0001

Supplemental Table 3: Odds Ratios Probability of Mortality within 180 Days

	Odds Ratio	Lower CI	Upper CI	p-value
**Reference group no SSRI Rx in 180-day period prior to surgery**				
SSRI RX 180 days prior to surgery	1.256	1.016	1.554	0.0351
**Reference year 2016**				
Year of surgery 2017	1.181	0.836	1.669	0.3454
Year of surgery 2018	1.177	0.840	1.65	0.3424
Year of surgery 2019	0.807	0.567	1.148	0.2334
Year of surgery 2020	1.048	0.747	1.469	0.7863
Year of surgery 2021	1.122	0.801	1.570	0.5035
**Reference age 65-84**				
Age < 65	0.664	0.411	1.072	0.0938
Age >= 85	1.917	1.560	2.354	<.0001
**Reference male**				
Female	0.538	0.436	0.664	<.0001
**Reference not diagnosed with condition**				
One Charlson co-morbidity	1.220	0.836	1.779	0.3018
Two or more Charlson co-morbidities	1.642	1.157	2.331	0.0056
Dementia diagnosis	2.955	2.396	3.645	<.0001
Depression diagnosis	0.809	0.635	1.030	0.085
Anemia diagnosis	1.279	1.02	1.604	0.0331
**Reference group median neigbourhood income 25th-75th percentile**				
Median neigbourhood income < 25th percentile	1.066	0.838	1.355	0.6037
Median neigbourhood income > 75th percentile	0.881	0.683	1.136	0.3284
**Reference distance from surgical hospital <=19**				
Lives 20-49 kms from surgical hospital	0.61	0.409	0.909	0.0152
Lives 50-100 kms from surgical hospital	0.95	0.720	1.253	0.7173
Lives > 100 kms from surgical hospital	0.912	0.660	1.261	0.5776
**Reference lives in urban area**				
Lives in a rural area	1.059	0.82	1.368	0.6608

Supplemental Table 4: Odds Ratios Probability of Revision within 180 Days

	Odds Ratio	Lower CI	Upper CI	p-value
**Reference group no SSRI Rx in 180-day period prior to surgery**				
SSRI RX 180 days prior to surgery	2.334	1.351	4.035	0.0024
**Reference year 2016**				
Year of surgery 2017	1.312	0.513	3.359	0.5707
Year of surgery 2018	1.442	0.585	3.555	0.4262
Year of surgery 2019	0.981	0.377	2.549	0.9679
Year of surgery 2020	1.085	0.421	2.795	0.8659
Year of surgery 2021	1.049	0.404	2.723	0.9219
**Reference age 65-84**				
Age < 65	1.061	0.463	2.429	0.8883
Age >= 85	0.273	0.126	0.591	0.001
**Reference male**				
Female	2.283	1.085	4.804	0.0297
**Reference not diagnosed with condition**				
One Charlson co-morbidity	8.221	1.364	49.54	0.0215
Two or more Charlson co-morbidities	4.949	0.824	29.741	0.0805
Dementia diagnosis	0.249	0.086	0.721	0.0104
Depression diagnosis	0.708	0.359	1.393	0.3165
Anemia diagnosis	1.283	0.690	2.388	0.4314
**Reference group median neigbourhood income 25th-75th percentile**				
Median neigbourhood income < 25th percentile	0.672	0.326	1.383	0.2805
Median neigbourhood income > 75th percentile	1.026	0.529	1.99	0.9403
**Reference distance from surgical hospital <=19**				
Lives 20-49 kms from surgical hospital	0.601	0.192	1.883	0.3825
Lives 50-100 kms from surgical hospital	1.554	0.771	3.132	0.2174
Lives > 100 kms from surgical hospital	1.002	0.392	2.563	0.9965
**Reference lives in urban area**				
Lives in a rural area	0.857	0.430	1.707	0.6599

Supplemental Table 5: Odds Ratios Probability of a Blood Transfusion

	Odds Ratio	Lower CI	Upper CI	p-value
**Reference group no SSRI Rx in 180-day period prior to surgery**				
SSRI RX 180 days prior to surgery	1.091	0.82	1.45	0.5495
**Reference year 2016**				
Year of surgery 2017	1.386	0.883	2.178	0.1562
Year of surgery 2018	1.223	0.778	1.924	0.3826
Year of surgery 2019	1.092	0.691	1.725	0.705
Year of surgery 2020	1.011	0.633	1.613	0.9649
Year of surgery 2021	1.429	0.912	2.238	0.1189
**Reference age 65-84**				
Age < 65	1.317	0.837	2.072	0.2339
Age >= 85	1.191	0.898	1.579	0.2249
**Reference male**				
Female	1.043	0.779	1.396	0.7765
**Reference not diagnosed with condition**				
One Charlson co-morbidity	1.137	0.56	2.312	0.7219
Two or more Charlson co-morbidities	1.981	1.030	3.809	0.0404
Dementia diagnosis	0.758	0.552	1.041	0.0871
Depression diagnosis	1.014	0.749	1.373	0.928
Anemia diagnosis	5.08	3.797	6.797	<.0001
**Reference group median neigbourhood income 25th-75th percentile**				
Median neigbourhood income < 25th percentile	0.893	0.641	1.243	0.502
Median neigbourhood income > 75th percentile	1.027	0.741	1.423	0.8745
**Reference distance from surgical hospital <=19**				
Lives 20-49 kms from surgical hospital	0.578	0.33	1.009	0.0539
Lives 50-100 kms from surgical hospital	0.615	0.382	0.989	0.0450
Lives > 100 kms from surgical hospital	1.359	0.852	2.168	0.1979
**Reference lives in urban area**				
Lives in a rural area	1.067	0.721	1.578	0.7467

Supplemental Table 6: Odds Ratios Probability of a Bleeding Event within 180 Days

	Odds Ratio	Lower CI	Upper CI	p-value
**Reference group no SSRI Rx in 180-day period prior to surgery**				
SSRI RX 180 days prior to surgery	1.479	1.058	2.068	0.0222
**Reference year 2016**				
Year of surgery 2017	1.215	0.653	2.261	0.5377
Year of surgery 2018	1.562	0.877	2.784	0.1303
Year of surgery 2019	1.278	0.710	2.303	0.4132
Year of surgery 2020	1.669	0.951	2.930	0.0742
Year of surgery 2021	1.433	0.800	2.566	0.2268
**Reference age 65-84**				
Age < 65	1.097	0.635	1.894	0.7409
Age >= 85	0.674	0.468	0.973	0.0349
**Reference male**				
Female	0.640	0.458	0.895	0.0091
**Reference not diagnosed with condition**				
One Charlson co-morbidity	1.452	0.690	3.057	0.3258
Two or more Charlson co-morbidities	2.516	1.270	4.984	0.0082
Dementia diagnosis	0.828	0.552	1.240	0.3593
Depression diagnosis	0.754	0.502	1.132	0.1731
Anemia diagnosis	1.154	0.807	1.651	0.433
**Reference group median neigbourhood income 25th-75th percentile**				
Median neigbourhood income < 25th percentile	0.983	0.649	1.491	0.9368
Median neigbourhood income > 75th percentile	1.463	0.996	2.148	0.0526
**Reference distance from surgical hospital <=19**				
Lives 20-49 kms from surgical hospital	0.747	0.394	1.416	0.3713
Lives 50-100 kms from surgical hospital	1.199	0.778	1.848	0.4109
Lives > 100 kms from surgical hospital	0.651	0.354	1.200	0.1690
**Reference lives in urban area**				
Lives in a rural area	0.900	0.585	1.385	0.6329

Supplemental Table 7: Odds Ratios Probability of an Emergency Department Visit within 90 Days

	Odds Ratio	Lower CI	Upper CI	p-value
**Reference group no SSRI Rx in 180-day period prior to surgery**				
SSRI RX 180 days prior to surgery	1.652	1.347	2.025	<.0001
**Reference year 2016**				
Year of surgery 2017	1.011	0.712	1.434	0.9517
Year of surgery 2018	1.365	0.982	1.897	0.0640
Year of surgery 2019	1.024	0.731	1.434	0.8921
Year of surgery 2020	1.411	1.024	1.944	0.0354
Year of surgery 2021	0.931	0.659	1.315	0.6849
**Reference age 65-84**				
Age < 65	1.368	0.985	1.899	0.0612
Age >= 85	0.861	0.694	1.067	0.1713
**Reference male**				
Female	0.856	0.691	1.06	0.1540
**Reference not diagnosed with condition**				
One Charlson co-morbidity	1.487	1.011	2.186	0.0438
Two or more Charlson co-morbidities	2.068	1.445	2.958	<.0001
Dementia diagnosis	0.598	0.465	0.769	<.0001
Depression diagnosis	1.076	0.854	1.356	0.5342
Anemia diagnosis	0.846	0.674	1.062	0.1498
**Reference group median neigbourhood income 25th-75th percentile**				
Median neigbourhood income < 25th percentile	1.098	0.867	1.391	0.4388
Median neigbourhood income > 75th percentile	0.847	0.659	1.088	0.1940
**Reference distance from surgical hospital <=19**				
Lives 20-49 kms from surgical hospital	0.729	0.498	1.068	0.1046
Lives 50-100 kms from surgical hospital	0.638	0.465	0.874	0.0052
Lives > 100 kms from surgical hospital	0.814	0.575	1.152	0.2459
**Reference lives in urban area**				
Lives in a rural area	0.651	0.499	0.849	0.0015

Supplemental Table 8: Odds Ratios Probability of Mortality within 90 Days

	Odds Ratio	Lower CI	Upper CI	p-value
**Reference group no SSRI Rx in 180-day period prior to surgery**				
SSRI RX 180 days prior to surgery	0.983	0.772	1.251	0.8874
**Reference year 2016**				
Year of surgery 2017	1.322	0.914	1.912	0.1388
Year of surgery 2018	0.889	0.605	1.305	0.5473
Year of surgery 2019	0.74	0.501	1.095	0.1317
Year of surgery 2020	1.015	0.701	1.47	0.9378
Year of surgery 2021	1.076	0.744	1.555	0.6964
**Reference age 65-84**				
Age < 65	0.686	0.4	1.175	0.1701
Age >= 85	1.879	1.496	2.361	<.0001
**Reference male**				
Female	0.566	0.449	0.714	<.0001
**Reference not diagnosed with condition**				
One Charlson co-morbidity	1.188	0.783	1.804	0.418
Two or more Charlson co-morbidities	1.469	0.995	2.167	0.0528
Dementia diagnosis	3.025	2.406	3.803	<.0001
Depression diagnosis	0.885	0.68	1.153	0.3665
Anemia diagnosis	1.377	1.074	1.766	0.0117
**Reference group median neigbourhood income 25th-75th percentile**				
Median neigbourhood income < 25th percentile	1.138	0.874	1.484	0.3373
Median neigbourhood income > 75th percentile	0.983	0.742	1.301	0.9027
**Reference distance from surgical hospital <=19**				
Lives 20-49 kms from surgical hospital	0.62	0.395	0.972	0.0373
Lives 50-100 kms from surgical hospital	0.956	0.702	1.302	0.7743
Lives > 100 kms from surgical hospital	1.036	0.727	1.475	0.8455
**Reference lives in urban area**				
Lives in a rural area	0.973	0.731	1.295	0.8492

Supplemental Table 9: Odds Ratios Probability of Revision within 90 Days

	Odds Ratio	Lower CI	Upper CI	p-value
**Reference group no SSRI Rx in 180-day period prior to surgery**				
SSRI RX 180 days prior to surgery	2.679	1.466	4.896	0.0014
**Reference year 2016**				
Year of surgery 2017	2.003	0.694	5.779	0.1989
Year of surgery 2018	1.881	0.655	5.402	0.2404
Year of surgery 2019	1.394	0.468	4.152	0.5509
Year of surgery 2020	1.346	0.443	4.089	0.6005
Year of surgery 2021	1.103	0.343	3.548	0.8690
**Reference age 65-84**				
Age < 65	1.278	0.52	3.142	0.5928
Age >= 85	0.287	0.126	0.656	0.0031
**Reference male**				
Female	2.74	1.157	6.489	0.0219
**Reference not diagnosed with condition**				
One Charlson co-morbidity	6.194	1.003	38.254	0.0496
Two or more Charlson co-morbidities	5.107	0.844	30.899	0.0758
Dementia diagnosis	0.341	0.116	1.001	0.0502
Depression diagnosis	0.483	0.208	1.12	0.0899
Anemia diagnosis	1.005	0.500	2.018	0.9899
**Reference group median neigbourhood income 25th-75th percentile**				
Median neigbourhood income < 25th percentile	0.576	0.249	1.328	0.1955
Median neigbourhood income > 75th percentile	1.126	0.548	2.316	0.7464
**Reference distance from surgical hospital <=19**				
Lives 20-49 kms from surgical hospital	0.804	0.247	2.614	0.7163
Lives 50-100 kms from surgical hospital	2.032	0.949	4.348	0.0678
Lives > 100 kms from surgical hospital	1.059	0.364	3.082	0.9156
**Reference lives in urban area**				
Lives in a rural area	0.939	0.448	1.968	0.8678

Supplemental Table 10: Odds Ratios Probability of a Bleeding Event within 90 Days

	Odds Ratio	Lower CI	Upper CI	p-value
**Reference group no SSRI Rx in 180-day period prior to surgery**				
SSRI RX 180 days prior to surgery	1.46	0.961	2.22	0.0763
**Reference year 2016**				
Year of surgery 2017	1.453	0.682	3.094	0.3330
Year of surgery 2018	1.769	0.867	3.609	0.1171
Year of surgery 2019	1.119	0.521	2.402	0.7732
Year of surgery 2020	2.002	1.006	3.981	0.0479
Year of surgery 2021	1.135	0.525	2.455	0.7470
**Reference age 65-84**				
Age < 65	1.062	0.542	2.081	0.8599
Age >= 85	0.497	0.307	0.804	0.0044
**Reference male**				
Female	0.6	0.396	0.909	0.0159
**Reference not diagnosed with condition**				
One Charlson co-morbidity	3.382	0.866	13.206	0.0795
Two or more Charlson co-morbidities	5.794	1.565	21.443	0.0085
Dementia diagnosis	0.873	0.524	1.455	0.6030
Depression diagnosis	0.697	0.418	1.164	0.1681
Anemia diagnosis	1.742	1.137	2.669	0.0108
**Reference group median neigbourhood income 25th-75th percentile**				
Median neigbourhood income < 25th percentile	1.086	0.651	1.81	0.7528
Median neigbourhood income > 75th percentile	1.501	0.922	2.443	0.1023
**Reference distance from surgical hospital <=19**				
Lives 20-49 kms from surgical hospital	1.010	0.490	2.083	0.9776
Lives 50-100 kms from surgical hospital	1.214	0.706	2.09	0.4827
Lives > 100 kms from surgical hospital	0.485	0.215	1.098	0.0827
**Reference lives in urban area**				
Lives in a rural area	1.328	0.790	2.23	0.2844
